# An ethanol root extract of *Cynanchum wilfordii* containing acetophenones suppresses the expression of VCAM-1 and ICAM-1 in TNF-α-stimulated human aortic smooth muscle cells through the NF-κB pathway

**DOI:** 10.3892/ijmm.2015.2112

**Published:** 2015-02-26

**Authors:** HYUN JUNG KOO, EUN-HWA SOHN, SUHKNEUNG PYO, HAN GOO WOO, DAE WON PARK, YOUNG-MIN HAM, SEON-A JANG, SOO-YEONG PARK, SE CHAN KANG

**Affiliations:** 1 Department of Medicinal and Industrial Crops, Korea National College of Agriculture and Fisheries, Jeonju 560-500, Republic of Korea; 2 Department of Herbal Medicine Resources, Kangwon National University, Samcheok, Gangwon-do 245-710, Republic of Korea; 3 Division of Immunopharmacology, School of Pharmacy, Sungkyunkwan University, Suwon, Gyeonggi-do 440-746, Republic of Korea; 4 Department of Life Science, Gachon University, Seongnam, Gyeonggi-Do 461-701, Republic of Korea; 5 Jeju Biodiversity Research Institute, Jeju Technopark, Jeju 699-943, Republic of Korea

**Keywords:** *Cynanchum wilfordii*, acetophenone, vascular inflammation, cell adhesion molecule, aorta

## Abstract

The root of *Cynanchum wilfordii* (*C. wilfordii*) contains several biologically active compounds which have been used as traditional medicines in Asia. In the present study, we evaluated the anti-inflammatory effects of an ethanol root extract of *C. wilfordii* (CWE) on tumor necrosis factor (TNF)-α-stimulated human aortic smooth muscle cells (HASMCs). The inhibitory effects of CWE on vascular cell adhesion molecule (VCAM)-1 expression under an optimum extraction condition were examined. CWE suppressed the expression of VCAM-1 and ICAM-1 and the adhesion of THP-1 monocytes to the TNF-α-stimulated HASMCs. Consistent with the *in vitro* observations, CWE inhibited the aortic expression of ICAM-1 and VCAM-1 in atherogenic diet-fed mice. CWE also downregulated the expression of nuclear factor-κB (NF-κB p65) and its uclear translocation in the stimulated HASMCs. In order to identify the active components in CWE, we re-extracted CWE using several solvents, and found that the ethyl acetate fraction was the most effective in suppressing the expression of VCAM-1 and ICAM-1. Four major acetophenones were purified from the ethyl acetate fraction, and two components, *p*-hydroxyacetophenone and cynandione A, potently inhibited the expression of ICAM-1 and VCAM-1 in the stimulated HASMCs. We assessed and determined the amounts of these two active components from CWE, and our results suggested that the root of *C. wilfordii* and its two bioactive acetophenones may be used for the prevention and treatment of atherosclerosis and vascular inflammatory diseases.

## Introduction

Vascular inflammation is a complex and multifactorial pathophysiological process that plays a key role in the development and progression of various cardiovascular diseases, including atherosclerosis and congestive heart failure (1,2). There are various risk factors involved, such as oxidative stress and modified low-density lipoprotein (LDL) cholesterol that may contribute to the onset and progression of vascular inflammation and result in chronic inflammation (3). This process is predominantly mediated by a diverse group of cell adhesion molecules (CAMs), which are expressed on the surface of vascular endothelial cells and smooth muscle cells in response to several inflammatory stimuli (4). The interaction between leukocytes and vascular cells is considered a hallmark of vascular inflammation (4,5). Indeed, clinical studies have demonstrated that the increased expression of CAMs, such as intercellular adhesion molecule-1 (ICAM-1) and vascular cell adhesion molecule-1 (VCAM-1) contributes to vascular dysfunction through the recruitment of inflammatory cells and their transmigration into target sites. Therefore, the functional inhibition of CAMs may be a critical therapeutic strategy for the treatment of vascular diseases.

During vascular inflammation, pro-inflammatory cytokines, such as tumor necrosis factor (TNF)-α, C-reactive protein and interleukin (IL)-6 appear to accelerate vascular dysfunction by inducing the expression of CAMs, which leads to the alternation of cell-cell and cell-matrix interactions (6). In particular, TNF-α has been implicated as a central mediator of vascular inflammation (7). TNF-α causes vascular oxidative stress, vascular remodeling, thrombosis, cell infiltration and apoptosis and leads to vascular damage (8,9). Therefore, in the present study, we used TNF-α to induce vascular inflammation in human aortic smooth muscle cells (HASMCs).

The root of *Cynanchum wilfordii* (*C. wilfordii*) has been used widely as a traditional herbal medicine in Asia for the treatment of insomnia, anxiety, anemia, senescence and various geriatric diseases. The biological effects of the root of *C. wilfordii* against tumors, antioxidants, diabetes mellitus, gastric disorders, neuronal damage and hypercholesterolemia have been reported (10–15).

However, there is little information available on the molecular mechanisms responsible for the anti-inflammatory effects of the extract and bioactive components of the root of *C. wilfordii* on vascular-type cells. It is known that the root of *C. wilfordii* contains several active compounds, including gagaminine, pregnane glycosides, cynanchone, various wilfosides and cynauricuosides, sarcotine, penupogenin, cynandione A (Cyn A) and anthraquinones (16). Recently, Yang *et al* (17) reported that Cyn A from the root of *C. wilfordii* exerts anti-inflammatory effects on lipopolysaccharide-treated brain macrophages/BV2 microglial cells.

In the present study, we investigated the anti-inflammatory effects of a root extract of *C. wilfordii* under optimal extraction conditions in order to elucidate the molecular mechanisms of action of the vascular protective properties of the root of *C. wilfordii* and identify its major active components.

## Materials and methods

### Materials and reagents

The chemicals used in the present study were purchased from Sigma-Aldrich (St. Louis, MO, USA). Antibodies against ICAM-1 (Cat. no. 4915), p65 (Cat. no. 8242), lamin A/C (Cat. no. 2032) and β-actin (Cat. no. 4967) were obtained from Cell Signaling Technology (Beverly, MA, USA). Anti-VCAM-1 antibody (sc-8304) was purchased from Santa Cruz Biotechnology (Santa Cruz, CA, USA).

### Cell culture

Primary HASMCs were obtained from ScienCell Research Laboratories (San Diego, CA, USA). The cells were cultured as monolayers in smooth muscle cell (SMC) medium (ScienCell) containing essential and non-essential amino acids, vitamins, organic and inorganic compounds, hormones, growth factors, trace minerals and 2% fetal bovine serum (FBS) at 37°C in a humidified atmosphere of 95% air and 5% CO_2_. For subcultures, the cells were detached using 0.125% trypsin containing 0.01 M ethylenediaminetetraacetic acid (EDTA). The cells used in the present study were from the early passages (passages 2–6). THP-1 cells were from the American Type Culture Collection (ATCC; Manassas, VA, USA) and weres used for the cell adhesion assay with the HASMCs. These cells were cultured in RPMI-1640, and supplemented with 2 mM L-glutamine, 100 mg/ml streptomycin, 100 IU/ml penicillin and 10% FBS.

### Preparation and characterization of an ethanol root extract of C. wilfordii (CWE)

The root of C. *wilfordii* used in the present study was collected through KNRRC (Medicinal Plants Resources Bank NRF-2010-0005790) supported by the Korea Research Foundation (the resources of which were provided by the Ministry of Education, Science and Technology of Korea) in 2014. A voucher specimen (no. MPRBP00962) was deposited in the herbarium of Gachon University (Seongnam, Korea). The powder from the root of *C. wilfordii* (2,500 g) was extracted twice with 0–100% ethanol for 48 h at room temperature and the extract was concentrated under reduced pressure. The decoction was filtered, lyophilized and stored at 4°C until use.

### Isolation and structural identification of components from CWE

CWE (100 g) was dissolved in distilled water and partitioned with *n*-hexane (Hx fraction), dichloromethane (CH_2_Cl_2_, MC fraction), ethyl acetate (EtOAc fraction), *n*-butanol (*n*-BuOH fraction) and water (H_2_O fraction). The yield of dried extract from the starting crude materials was approximately 30.8% (wt/wt). The EtOAc fraction has a potent suppressive effect on the expression of the adhesion molecules, VCAM-1 and ICAM-1. Hence, the EtOAc fraction (2.55 g) was fixed on Celite, fractionated by vacuum liquid chromatography (VLC) on a silica gel, and eluted to 16 sub-fractions. Fractions 6, 8, 10 and 12 were subjected to silica gel column chromatography (CC) (150 g, 50×120 mm) and eluted with hexane/EtOAc/methanol (3:1:0.3) to yield 2,4-dihydroxyacetophenone (2,4-DHA), 2,5-dihydroxyacetophenone (2,5-DHA), *p*-hydroxyacetophenone (*p*-HA) and (Cyn A):

- 2,4-DHA: colorless solid; ^1^H-NMR (500 MHz, CDCl_3_): *δ* 7.72 (1H, d, *J*=7.04 Hz), 6.35 (1H, dd, *J*=2.5 and 7.04 Hz), 6.24 (1H, d, *J*=2.0 Hz), 2.51 (3H, s); ^13^C-NMR (125 MHz, CDCl_3_): *δ* 114.5, 166.6, 103.6, 166.4, 109.2, 134.6, 204.3, 26.3. ii) 2,5-DHA: yellow powder; ^1^H-NMR (500 MHz, CDCl_3_): *δ* 7.21 (1H, d, *J*=2.24 Hz), 7.01 (1H, dd, *J*=2.5 and 7.04 Hz), 6.78 (1H, d, *J*=7.3 Hz), 2.58 (3H, s); ^13^C-NMR (125 MHz, CDCl_3_): *δ* 120.8, 156.7, 119.7, 166.4, 126.0, 116.5, 206.0, 27.0.
- *p*-HA: colorless powder; ^1^H-NMR (500 MHz, CDCl_3_): *δ* 7.89 (2H, d, *J*=8.92 Hz), 6.84 (2H, d, *J*=10.12 Hz), 2.51 (3H, s); ^13^C-NMR (125 MHz, CDCl_3_): *δ* 130.2, 132.2, 116.2, 164.0, 199.5, 26.3.
- Cyn A: yellow needles; ^1^H-NMR (500 MHz, CDCl_3_): *δ* 6.94 (1H, d, *J*=8.92 Hz), 6.80 (1H, d, *J*=8.92 Hz), 6.50 (1H, d, *J*=8.96 Hz), 2.57 (3H, s), 2.17 (3H, s); ^13^C-NMR (125 MHz, CDCl_3_): *δ* 127.8, 120.4, 152.4, 118.2, 121.8, 149.1, 207.4, 31.0, 114.5, 163.8, 113.2, 134.0, 108.8, 163.7, 204.6, 26.4.

### Experimental animals

The experimental animal facility and study protocols (GIACUC-R2013017) were approved by the Animal Care and Use Committee of Gachon University. All experimental procedures were undertaken in compliance with the Guide for the Care and Use of Laboratory Animals (National Institutes of Health, Bethesda, MD, USA) and the National Animal Welfare Law of the Republic of Korea.

Four-week-old male C57BL/6 mice were obtained from Japan SLC Inc. (Shizuoka, Japan) and maintained in a controlled environment of 22±1°C and a humidity of 50±10% with a 12-h light-dark cycle for 1 week prior to the commencement of the experiments. Mice had access to sterile standard mouse chow and water *ad libitum*. At the start of the study, the diet was changed to an atherogenic (ATH) diet [1.25% (w/w) cholesterol, 0.5% (w/w) cholic acid and 16% (w/w) fats in the form of soybean oil, cocoa butter and coconut oil] or a normal control (NC) chow [0.3% (w/w) cholesterol, no cholic acid and 5% (w/w) fats]. Both diets were obtained from Research Diets Inc. (New Brunswick, NJ, USA).

The mice were divided randomly into 6 groups of 5 mice as follows: i) mice fed a normal control chow diet plus the vehicle (PBS; NC group); ii) mice fed an ATH diet plus the vehicle (PBS; ATH group); iii) mice fed an ATH diet plus 50 mg/kg body weight (bw)/day of CWE; iv) mice fed an ATH diet plus 100 mg/kg bw/day of CWE; v) mice fed an ATH diet plus 200 mg/kg bw/day of CWE; and vi) mice fed an ATH diet plus 10 mg/kg bw/day of simvastatin (Simv; Sigma-Aldrich) via oral gavage for 12 weeks. At the end of the treatments, each mouse was anesthetized and the thorax was opened. The aorta was dissected following perfusion with phosphate-buffered saline (PBS) and stored at −80°C until RNA isolation.

### Cell viability

The HASMCs were seeded in 96-well flat-bottom plates (2×10^4^ cells/well) and then treated with CWE (2, 20 and 200 *μ*g/ml) for 16 h. The cells were incubated with 100 *μ*l of 5 mg/ml MTT [3-(4,5-dimethylthiazolyl)-2,5-diphenyl-tetrazolium bromide] (Sigma-Aldrich) for a further 2–4 h. After the supernatant was removed, 100 *μ*l of DMSO per well was added to the cells and mixed on a shaker for 15 min to dissolve the formazan crystals formed. The optical density (OD) colored solution was quantified at a 570 nm wavelength using an enzyme-linked immunoabsorbent assay (ELISA) reader (model 550 microplate reader, Bio-Rad Laboratories, Hercules, CA, USA).

### Monocyte adhesion assay

The adhesion of THP-1 cells to the HASMCs was measured as previously described (18). Briefly, the HASMCs (which were grown in 96-well plates) were pre-treated with CWE (2, 20 and 200 *μ*g/ml) for 2 h at 37°C. The cells were washed with medium and incubated with fresh growth medium containing TNF-α (10 ng/ml) for 8 h. The medium was removed from the wells and calcein AM-labeled THP-1 cells (2×10^5^ cells/ml) in 0.2 ml of the medium were added to each well. The test and control samples were used in triplicate in each experiment. Following incubation for 1 h in 5% CO_2_ at 37°C, micro-wells were washed twice with 0.2 ml of warm medium. The number of adherent cells was detected using a fluorescence microscope (IX71; Olympus, Tokyo, Japan) equipped with a digital camera (DP71; Olympus) and processed using ImageJ software version 1.45s (National Institutes of Health). The increase in the adhesion of THP-1 cells upon stimulation of the HASMCs with TNF-α was calculated in relation to the basal adhesion of THP-1 cells to the unstimulated HASMCs (which was set to 1).

### Western blot analysis

The cells were pretreated with CWE (2, 20 and 200 *μ*g/ml) or dexamethasone (50 ng/ml) for 2 h. The cells were washed with medium and incubated with fresh growth medium containing TNF-α (10 ng/ml) for 30 min or 8 h. Following treatment, the cells were washed twice with PBS and lysed in ice-cold lysis buffer [50 mM Tris-HCl (pH 7.4), 150 mM NaCl, 1 mM EDTA, 0.5% (v/v) NP-40, 0.1% (w/v) sodium dodecyl sulfate (SDS)] containing protease inhibitor cocktail (Roche Diagnostics Corp., Indianapolis, IN, USA) for 1 h. The lysates were then collected after centrifugation at 1500 × g for 10 min at 4°C. Cytosolic and nuclear extracts were prepared using a Nuclear Extract kit (Active Motif, Carlsbad, CA, USA) according to the manufacturer’s instructions. The protein concentration was determined using a protein assay kit (Bio-Rad Laboratories) with bovine serum albumin (BSA) as the standard. Protein lysates (20 *μ*g) were subjected to 10% sodium dodecyl sulfate-polyacrylamide gel electrophoresis and transferred by electrophoretic means to an Immobilon^®^-P Polyvinylidene difluoride membrane (Amersham, Arlington Heights, IL, USA) and probed with appropriate antibodies. The blots were developed using an enhanced chemoluminescence (ECL) kit (Amersham). In all the westernt blotting experiments, the blots were re-probed with anti-β-actin antibody as a control for protein loading.

### RNA isolation and reverse transcription-polymerase chain reaction (RT-PCR)

Total RNA was extracted using a single-step guanidinium thiocyanate-phenol-chloroform method. The yield and purity of the RNA were confirmed by measuring the ratio of the absorbance values at 260 and 280 nm. PCR was undertaken using ICAM-1- and VCAM-1-specific primers to identify their respective specific cDNA. The following sequence-specific primers were synthesized: 5′-ATTTTCTGG GGCAGGAAGTT-3′ and 5′-ACGTCAGAACAACCGAAT CC-3′ for human VCAM-1; 5′-AGCACCTCCCCACCTAC TTT-3′ and 5′-AGCTTGCACGACCCTTCTAA-3′ for human ICAM-1. The following pair of oligonucleotides was used as the internal control: 5′-AACTTTGGCATTGTGGAAGG-3′ and 5′-ACACATGGGGGTAGGAACA-3′ for human glyceraldehyde-3-phosphate dehydrogenase (GAPDH). The absence of contaminants was routinely checked by an RT-PCR assay of negative control samples without the addition of a primer. Following amplification, the samples were stored at −20°C.

### High-performance liquid chromatography (HPLC)

An Alliance 2695 system (Waters Corp., Milford, MA, USA) coupled with a Waters 2998 photodiode array detector was used for the quantitative chromatographic analysis of CWE. The analytical column was a Sunfire™ 4.6 × 150 mm C18 column (particle size, 5 *μ*m; Waters Corp.). The mobile phase consisted of (A) acetic acid (0.5% v/v) and (B) acetonitrile using a gradient elution of A/B = 90/10 (0 min) → A/B = 65/35 (10 min) → A/B = 0/100 (30 min). The flow rate was 1.0 ml/min and the injection volume was 10 *μ*l; ultraviolet (UV) detection was conducted at 254 nm. CWE was dissolved in ethanol at 10 mg/ml, and *p*-HA (purity 99%), 2,4-DHA (purity 99%) and 2,5-DHA (purity 97%) were used as standard solutions.

### Statistical analysis

Each result is reported as the mean ± SEM. One-way analysis of variance was used to determine significance among groups, after which the modified Student’s t-test with the Bonferroni correction was used for the comparison between individual groups. A value of P<0.05 was considered to indicate a statistically significant difference.

## Results

### Optimal ethanol concentration for the root extract of C. wilfordii for the inhibition of the expression of adhesion molecules

**Figure 1:**
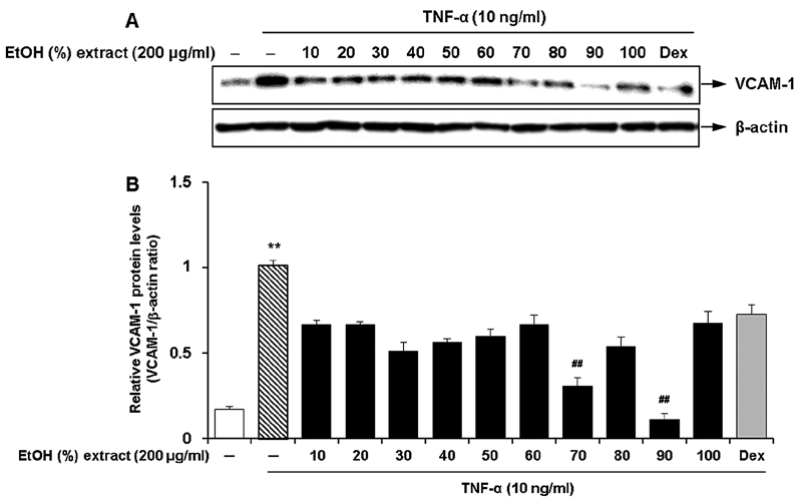
Effects of the ethanol concentration (10, 20, 30, 40, 50, 60, 70, 80, 90 and 100%, v/v) for the *Cynanchum wilfordii* extract (CWE) on the expression of vascular cell adhesion molecule (VCAM)-1 in tumor necrosis factor (TNF)-α-stimulated human aortic smooth muscle cells (HASMCs). (A) The cells were pre-treated with samples of each extract (200 *μ*g/ml) or dexamethasone (Dex; 50 ng/ml) for 2 h and then stimulated with TNF-α (10 ng/ml) for 12 h. The protein levels of VCAM-1 were determined by western blot analysis. (B) Densitometric analysis of western blots is represented as the mean band density normalized to β-actin. Results are the means ± SEM (n=3). Significantly different values are represented by a symbols (^**^P<0.01 compared to untreated control, ^##^P<0.01 compared to treatment with TNF-α alone).

We investigated the effects of the ethanol concentration for the root extract of *C. wilfordii* on the inhibition of the expression of adhesion molecules in the TNF-α-stimulated HASMCs. Ten different concentrations of ethanol (10, 20, 30, 40, 50, 60, 70, 80, 90 and 100%, v/v) were used by adjusting the composition of ethanol and water in the extraction solvent. The cells were pre-treated with 100 *μ*g/ml of each ethanol extract and then incubated with fresh growth medium containing TNF-α (10 ng/ml) for 8 h. We found that the ethanol extracts obtained with various concentrations had suppressive effects on TNF-α-induced VCAM-1 expression in the HASMCs (Fig. 1). Among these, the most marked inhibitory effect on VCAM-1 expression was observed by treatment with an ethanol concentration of 90% (Fig. 1). The extracts obtained at low or high ethanol concentrations (10, 20 and 100%) had lower efficacy, but they were comparable with the cells treated with dexamethasone (50 ng/ml).

### Inhibitory effect of CWE on the TNF-α-induced expression of adhesion molecules in HASMCs

**Figure 2:**
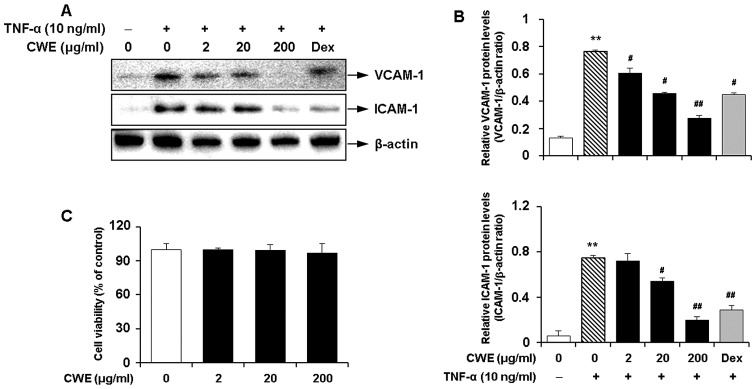
Inhibitory effects of a 90% ethanol extract of *Cynanchum wilfordii* (CWE) on the expression of cell adhesion molecules and the viability of human aortic smooth muscle cells (HASMCs). (A) HASMCs were pre-treated with CWE (2, 20 and 200 *μ*g/ml) for 2 h and then stimulated with tumor necrosis factor (TNF)-α (10 ng/ml) for 12 h. The protein expression of intercellular adhesion molecule-1 (ICAM-1) and vascular cell adhesion molecule (VCAM)-1 was determined by western blot analysis. (B) Densitometric analysis of western blots is represented as the mean band density normalized to β-actin. Results are the means ± SEM (n=3). Significantly different values are represented by symbols (^**^P<0.01 compared to untreated control, ^#^P<0.05, ^##^P<0.01 compared to treatment with TNF-α alone). (C) Cells at 80% confluence in 96-well plates were treated with CWE (2, 20 and 200 *μ*g/ml) for 16 h, and cell viability was determined by MTT assay. Values are the means ± SEM of triplicate experiments.

We determined the effects of CWE on the TNF-α-induced expression of adhesion molecules in HASMCs. Western blot analysis produced the following results i) TNF-α significantly induced the expression of VCAM-1 and ICAM-1; and ii) CWE downregulated the TNF-α-induced expression of the adhesion molecules in a dose-dependent manner (Fig. 2A and B).

Moreover, MTT assay revealed that CWE did not affect cell viability and was not cytotoxic to the cells at the concentrations used (Fig. 2C).

### Effect of CWE on the TNF-α-induced adhesion of THP-1 monocytes to HASMCs

**Figure 3:**
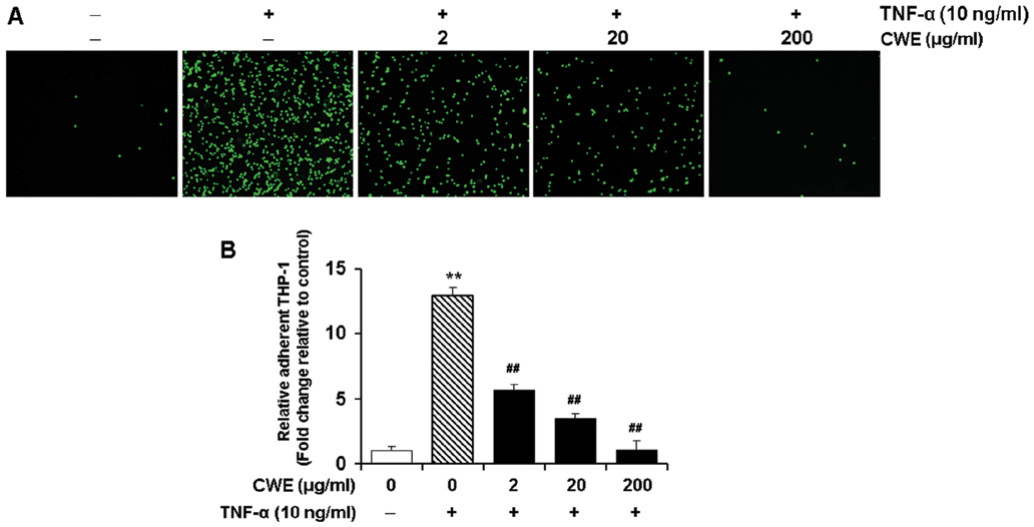
Effects of a 90% ethanol extract of *Cynanchum wilfordii* (CWE) on the adhesion of THP-1 cells to tumor necrosis factor (TNF)-α-stimulated human aortic smooth muscle cells (HASMCs). Confluent HASMCs were pre-treated for 2 h with CWE (2, 20 and 200 *μ*g/ml) and then incubated with TNF-α (10 ng/ml) for 12 h. (A) Calcein AM-labeled THP-1 cells were added to the HASMC monolayer and allowed to adhere for 1 h. The adherence of labeled THP-1 cells to HASMCs was observed under a fluorescence microscope at x100 magnification. (B) Values are the means ± SEM of triplicate experiments. Significantly different values are represented by symbols (^**^P<0.01 compared to untreated control, ^##^P<0.01 compared to treatment with TNF-α alone).

We determined the effects of CWE on the adherence of THP-1 monocytes to TNF-α-stimulated HASMCs. The HASMCs were treated without or with various concentrations (2, 20 and 200 *μ*g/ml) of CWE for 2 h prior to stimulation with TNF-α (10 ng/ml). Stimulation with TNF-α elicited a significant increase in the adhesion of THP-1 monocytes to the HASMCs (P<0.01). Treatment with CWE significantly inhibited the adhesion of the THP-1 monocytes to the HASMCs in a dose-dependent manner (Fig. 3).

### Effect of CWE on the expression of adhesion molecules in the aorta in vivo

**Figure 4:**
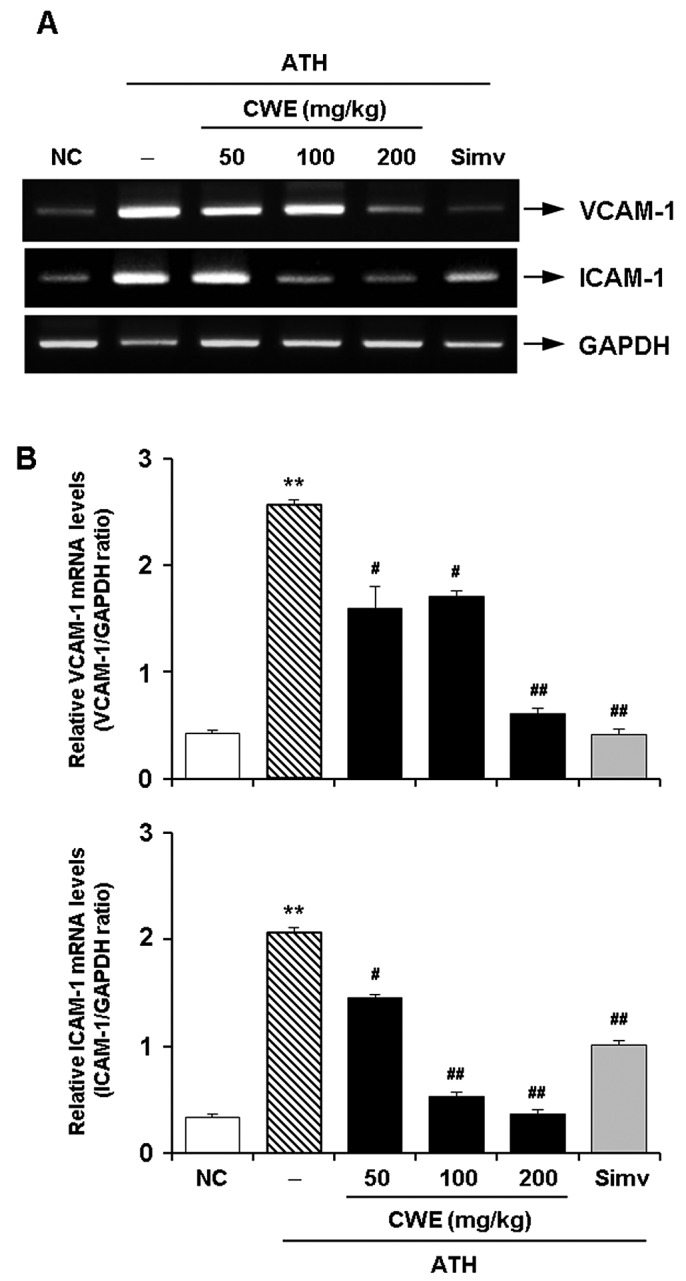
Effect of 90% ethanol extract of *Cynanchum wilfordii* (CWE) on the aortic expression of cell adhesion molecules in mice fed an atherogenic (ATH) diet for 12 weeks. (A) Mice were orally administered 50, 100 and 200 mg/kg body weight (bw(/day of CWE or 10 mg/kg bw/day of simvastatin (Simv) along with an ATH diet for 12 weeks. The aorta was removed and the mRNA expression of intercellular adhesion molecule-1 (ICAM-1) and vascular cell adhesion molecule (VCAM)-1 was determined by RT-PCR. (B) Densitometric analysis of RT-PCR is represented as the mean band density normalized to GAPDH. Results are the means ± SEM (n=3). Significantly different values are represented by a symbols [^**^P<0.01 compared to normal chow diet (NC) control group, ^#^P<0.05, ^##^P<0.01 compared to an ATH diet (control group)].

To verify the *in vitro* effects of CWE, an *in vivo* experiment was undertaken using a mouse model of ATH diet-induced hypercholesterolemia. RT-PCR revealed the expected significant increase in the mRNA expression of VCAM-1 and ICAM-1 in the aortas of the hypercholesterolemic mice. The administration of CWE for 12 weeks dose-dependently reduced the expression of VCAM-1 and ICAM-1 in the aortaso the hypercholesterolemic mice. The suppressive effects of CWE (100 and 200 mg/kg) on the expression of CAMs were comparable to those observed by treatment with Simv (Fig. 4).

### Effect of CWE on the TNF-α-induced nuclear translocation of nuclear factor-κB (NF-κB)

**Figure 5:**
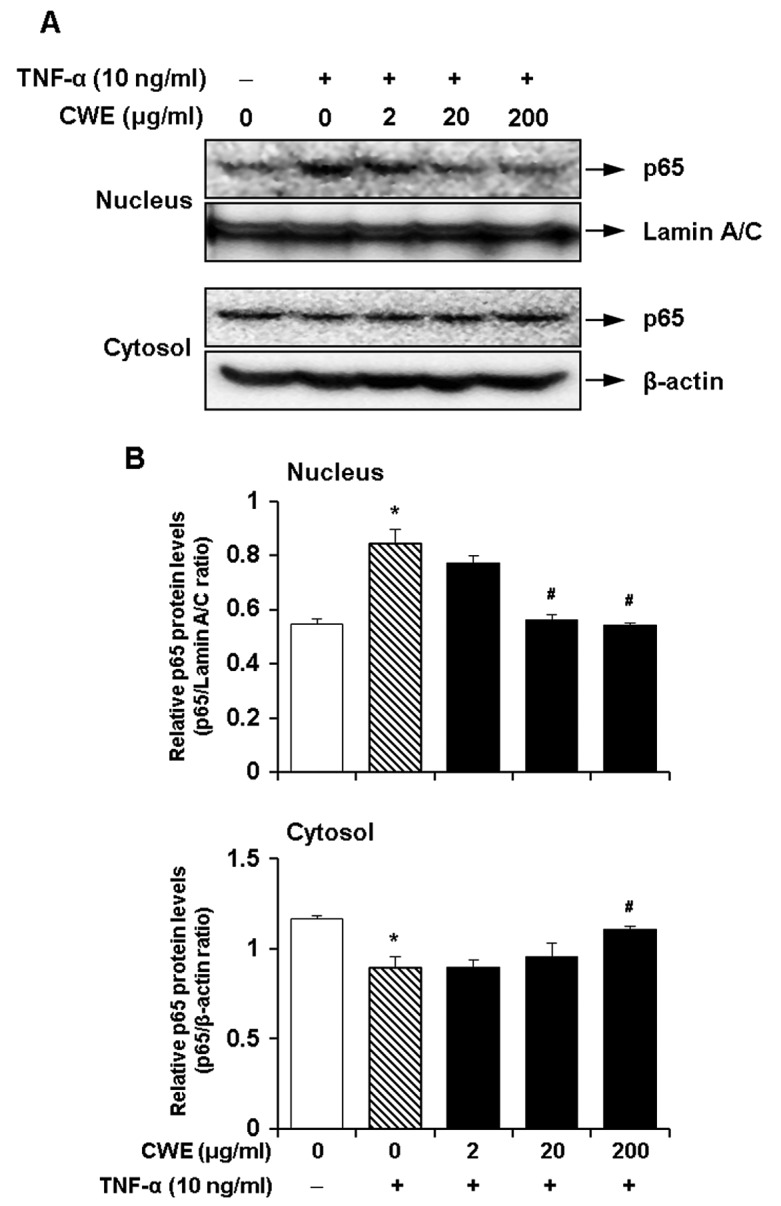
Effects of a 90% ethanol extract of *Cynanchum wilfordii* (CWE) on the nuclear translocation of the p65 subunit of nuclear factor (NF)-κB in tumor necrosis factor (TNF(-α-stimulated human aortic smooth muscle cells (HASMCs). (A) Cells were pre-incubated with CWE (2, 20 and 200 *μ*g/ml) for 2 h and then treated with TNF-α (10 ng/ml) for 30 min. Cytoplasmic and nuclear levels of p65 were determined by western blot analysis. (B) Densitometric analysis of the western blots is represented as the mean band density normalized to β-actin and Lamin A. Values are the means ± SEM (n=3). Significantly different values are represented by symbols (^*^P<0.05 compared to untreated control, ^#^P<0.05 compared to treatment with TNF-α alone).

NF-κB is a crucial transcription factor for the induction of the expression of adhesion molecules by TNF-α (19,20). Therefore, we investigated whether the inhibitory effects of CWE on the TNF-α-induced expression of ICAM-1 and VCAM-1 are mediated by the activation of NF-κB. The cells were treated with CWE (2, 20 and 200 *μ*g/ml) for 2 h prior to stimulation with TNF-α for 30 min. CWE decreased the translocation of NF-κB p65 to the nuclear fraction in a dose-dependent manner (Fig. 5). These data suggested that CWE inhibited the TNF-α-induced nuclear translocation of NF-κB.

### Effects of solvent fractions of CWE on the TNF-α-induced expression of adhesion molecules in HASMCs

**Figure 6:**
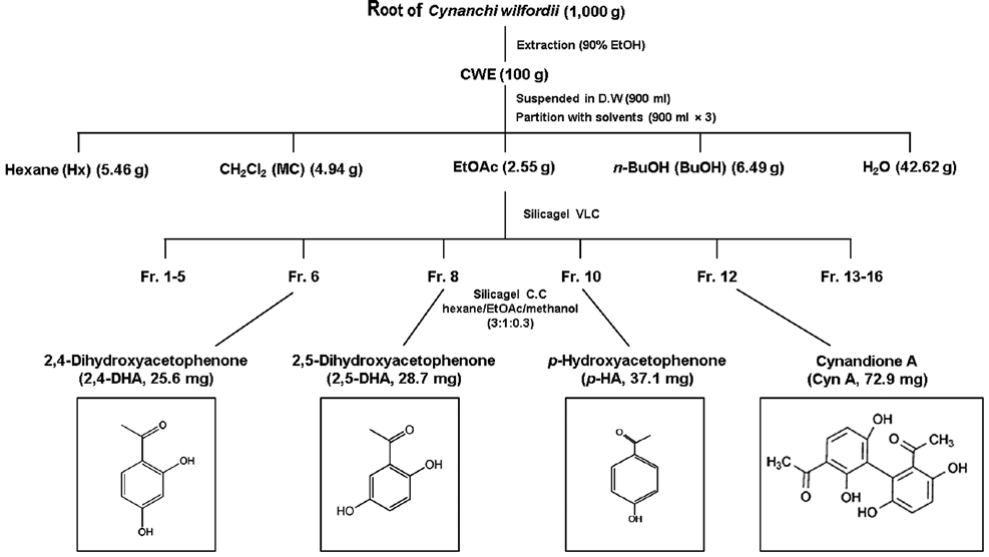
Extraction and fractionation of root extract *Cynanchum wilfordii* (schematic diagram). CH_2_Cl_2_, dichloromethane-soluble fraction; EtOAc, ethyl acetate-soluble fraction; *n*-BuOH, butanol-soluble fraction; H_2_O, water-soluble fraction.

**Figure 7:**
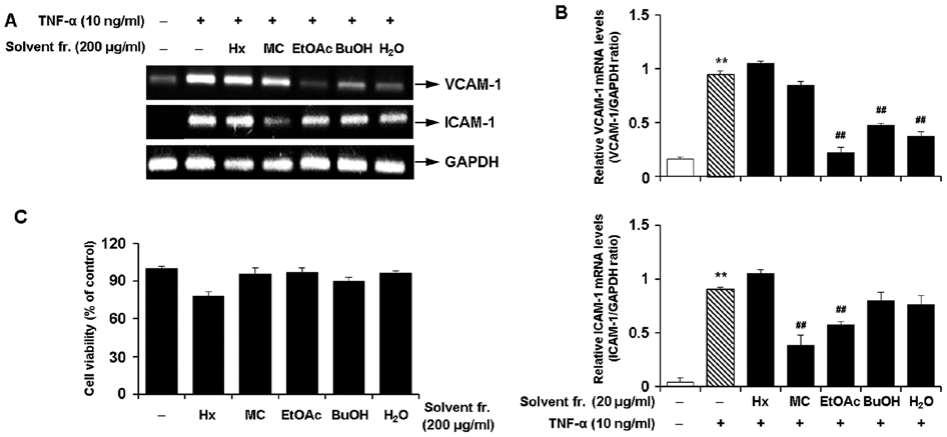
Effects of solvent fractions of *Cynanchum wilfordii* (CWE) on the expression of cell adhesion molecules in tumor necrosis factor (TNF)-α-stimulated human aortic smooth muscle cells (HASMCs). (A) The cells were pre-treated with solvent fractions (200 *μ*g/ml) for 2 h and then stimulated with TNF-α (10 ng/ml) for 12 h. The mRNA expression levels of intercellular adhesion molecule-1 (ICAM-1) and vascular cell adhesion molecule (VCAM)-1 were determined by RT-PCR. (B) Densitometric analysis of RT-PCR is represented as the mean band density normalized to GAPDH. Results are the means ± SEM (n=3). Significantly different values are represented by symbols (^**^P<0.01 compared to untreated control, ^##^P<0.01 compared to treatment with TNF-α alone). (C) Cells at 80% confluence in 96-well plates were treated with solvent fractions (200 *μ*g/ml) for 16 h, and cell viability was determined by MTT assay. Values are the means ± SEM of triplicate experiments.

We performed solvent fractionation of CWE and evaluated the effects of the fractions on the expression of adhesion molecules to select the most promising fraction (Fig. 6). RT-PCR revealed that the fraction with ethyl acetate (EtOAc) inhibited the mRNA expression of VCAM-1 and ICAM-1 by approximately 80 and 40% in the TNF-α-stimulated HASMCs, respectively (Fig. 7A and B). The EtOAc fraction was found to be more active with lower cytotoxicity (Fig. 7C) than the other fractions.

### Effect of the EtOAc fraction of CWE on the TNF-α-induced expression of adhesion molecules in HASMCs

**Figure 8:**
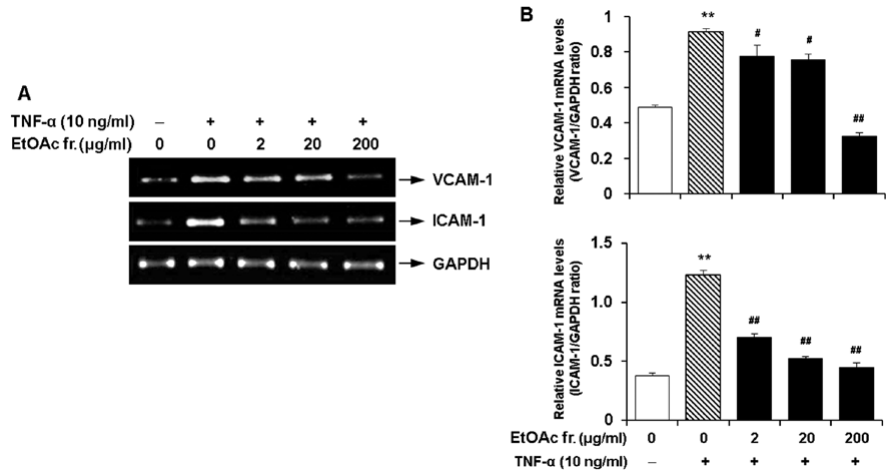
Inhibitory effects of different concentrations of the EtOAc fraction of *Cynanchum wilfordii* extract (CWE) on the expression of cell adhesion molecules in tumor necrosis factor (TNF)-α-stimulated human aortic smooth muscle cells (HASMCs). (A) Cells were pre-treated with the EtOAc fraction (2, 20 and 200 *μ*g/ml) for 2 h and then stimulated with TNF-α (10 ng/ml) for 12 h. The mRNA expression levels of intercellular adhesion molecule-1 (ICAM-1) and vascular cell adhesion molecule (VCAM)-1 were determined by RT-PCR. (B) Densitometric analysis of RT-PCR is represented as the mean band density normalized to GAPDH. Results are the means ± SEM (n=3). Significantly different values are represented by symbols (^**^P<0.01 compared to untreated control, ^#^P<0.05, ^##^P<0.01 compared to treatment with TNF-α alone).

As described above, the EtOAc fraction had the maximum inhibitory effect on the expression of VCAM-1 and ICAM-1 and did not elicit cytotoxicity. Hence, we investigated the dose-response effects of this fraction on the expression of VCAM-1 and ICAM-1 in the TNF-α-stimulated HASMCs. The EtOAc fraction markedly inhibited the TNF-α-induced mRNA expression of VCAM-1 and ICAM-1 in a dose-dependent manner (Fig. 8).

### Inhibitory effects of the major components of the EtOAc fraction of CWE on the TNF-α-induced expression of adhesion molecules in HASMCs

**Figure 9:**
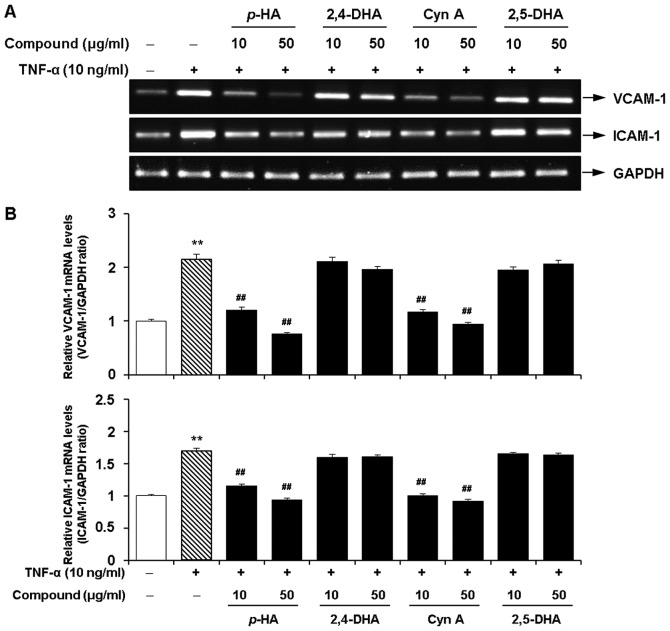
Effects of the 4 major acetophenones, *p*-hydroxyacetophenone (*p*-HA), 2,4-dihydroxyacetophenone (2,4-DHA), cynandione A (Cyn A) and 2,5-dihydroxyacetophenone (2,5-DHA), from the EtOAc fraction of *Cynanchum wilfordii* extract (CWE) on the expression of cell adhesion molecules in tumor necrosis factor (TNF)-α-stimulated human aortic smooth muscle cells (HASMCs). (A) Cells were pre-treated with 10 and 50 *μ*g/ml of each acetophenone for 2 h and then stimulated with TNF-α (10 ng/ml) for 12 h. The mRNA expression levels of intercellular adhesion molecule-1 (ICAM-1) and vascular cell adhesion molecule (VCAM)-1 were determined by RT-PCR. (B) Densitometric analysis of RT-PCR is represented as the mean band density normalized to GAPDH. Results are the means ± SEM (n=3). Significantly different values are represented by symbols (^**^P<0.01 compared to untreated control, ^##^P<0.01 compared to treatment with TNF-α alone).

Next, we investigated whether 4 major acetophenones from the EtOAc fraction of CWE (*p*-HA, 2,4-DHA, Cyn A and 2,5-DHA) inhibit the TNF-α-induced expression of VCAM-1 and ICAM-1 in the HASMCs. Among these components, *p*-HA and Cyn A significantly inhibited the mRNA expression of VCAM-1 and ICAM-1 at 10 and 50 *μ*g/ml (Fig. 9). However, treatment with 2,4-DHA and 2,5-DHA had little or no effect on the expression of VCAM-1 and ICAM-1. These 4 components did not affect cell viability at the concentrations tested (data not shown).

### Quantitative analysis of CWE

**Figure 10:**
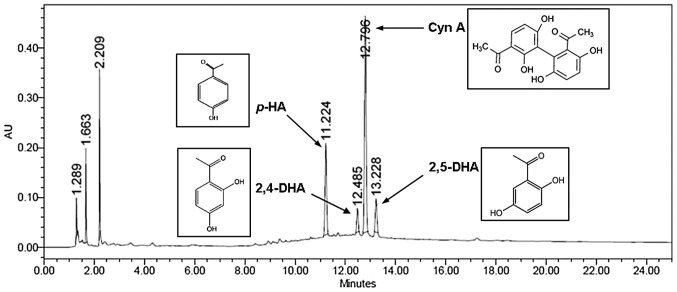
Representative chromatogram and UV spectrum of the 90% ethanol extract of *Cynanchum wilfordii* at 254 nm.

We applied HPLC for the simultaneous quantification of *p*-HA, 2,4-DHA, Cyn A and 2,5-DHA in CWE. The levels of *p*-HA, 2,4-DHA, Cyn A and 2,5-DHA identified at the retention times of 11.22, 12.49, 12.80 and 13.23 min were 3.8, 4.0, 21.0 and 1.0 mg/g, respectively (Fig. 10).

## Discussion

The arterial media comprises mainly of vascular smooth muscle cells (VSMCs). VSMCs contribute to the response to environ mental stresses and repair of the walls of blood vessels from vascular injury (21–23). In the vascular inflammatory reaction, the interactions of VSMCs with monocytes via CAMs are crucial events (24–26). Sutides have demonstrated that interactions between transmigrated monocytes and VSMCs induce monocyte pro-coagulant activity, pro-inflammatory responses and vascular dysfunction (27,28). The strong expression of CAMs, such as VCAM-1 and ICAM-1 in VSMCs in atherosclerotic lesions can facilitate the accumulation of transmigrated leukocytes within the vascular walls (29). Therefore, the inhibition of these mediators may be a promising strategy for the prevention and treatment of vascular inflammatory diseases (29,30).

The present study demonstrated the anti-inflammatory effects of CWE in TNF-α-stimulated human aortic SMCs. During the extraction or preparation of natural products, organic solvents, such as ethanol, methanol, acetone, ethyl acetate, dichloromethane or hexane are frequently used. Among these, ethanol is the most common completely biodegradable, edible and food-grade solvent (31). We selected the ethanol solvent and prepared various root extracts of *C. wilfordii* at an ethanol concentration range of 10 to 100%. We found that the 90% ethanol extract provided the optimal condition for the root of *C. wilfordii* to elicit the inhibition of VCAM-1 expression in the TNF-α-stimulated HASMCs. CWE inhibited the TNF-α-induced expression of VCAM-1 and ICAM-1 in the HASMCs in a dose-dependent manner.

Several studies have demonstrated that, in addition to endothelial cells, VSMCs also express ICAM-1 and VCAM-1 in atherosclerosis and vascular diseases (29,30). The expression of these molecules in VSMCs may facilitate the accumulation of transmigrated leukocytes within the vascular walls. It is well known that interactions between leukocytes and VSMCs can occur via CAMs, which can be antagonized by the inhibition of ICAM-1 and/or VCAM-1 (4). To confirm this hypothesis, we examined the effects of CWE on the monocyte THP-1 adherence to TNF-α-stimulated SMCs; we observed a marked reduction in monocyte adhesion in the CWE-treated groups in a dose-dependent manner.

We performed an animal experiment to confirm the suppressive effects of CWE on the expression of CAMs in the thoracic aortas of hypercholesterolemic mice. The administration of an ATH diet for 12 weeks resulted in the significantly increased expression of ICAM-1 and VCAM-1 in the aortic tissues. Several lines of evidence have suggested that exposure to a high-cholesterol diet potentiates systemic vascular inflammation, which leads to hypercholesterolemia and atherosclerosis. For example, Zhang *et al* (9) and Shi *et al* (32) demonstrated that the consumption of high-fat meals increases the plasma levels of TNF-α, IL-6, ICAM-1 and VCAM-1 and leads to vascular dysfunction. Our results clearly demonstrated that the administration of CWE downregulated the expression of ICAM-1 and VCAM-1 in ATH diet-fed mice.

NF-κB is an ubiquitous transcription factor crucial for the expression of inflammatory mediators (including CAMs) in VSMCs (33). It has been well established that NF-κB activation is associated with the nuclear translocation of the p65 component of the complex (34,35). We found that CWE inhibited the TNF-α-induced translocation of p65 to the nucleus. This finding suggests that the inhibitory effects of CWE on the expression of CAMs may be associated with the suppression of expression of NF-κB in VSMCs.

Several studies have demonstrated that active components from natural products can be converted into therapeutic agents (36–38). In a similar approach, we attempted to identify pharmacologically active components from CWE. CWE was fractionated with various solvents, and the EtOAc fraction showed maximal efficacy for the inhibition of the expression of VCAM-1 and ICAM-1 in the TNF-α-stimulated HASMCs. Subsequently, we performed further sub-fractionation and purification of the chemical components in the EtOAc fraction and identified 4 acetophenones: *p*-HA, 2,4-DHA, Cyn A and 2,5-DHA (Fig. 6).

Acetophenones are the major endogenous volatile organic compounds in plants. There is emerging evidence that acetophenones exert beneficial effects on vascular diseases. Ha *et al* (39) reported that acetophenones isolated from *Paeonia suffruticosa* Andr. stimulated the phosphorylation of endothelial nitric oxide synthase in human umbilical vein endothelial cells, which plays a role in vascular protection. Senejoux *et al* (40) also demonstrated that the naturally occurring acetophenone, apocynin, induced relaxation in aortic rings *in vitro* and reduced vascular pressure in spontaneously hypertensive rats. They demonstrated that apocynin exerted a vasorelaxant effect through the inhibition of the calcium ion-related contraction in VSMCs and the regulation of the production of endothelium-derived nitric oxide.

In the present study, we also investigated the anti-inflammatory effects of 4 acetophenones from CWE. We found that 2 components, *p*-hydroxyacetophenone and Cyn A, exerted suppressive effects on the expression of VCAM-1 and ICAM-1 in TNF-α-stimulated VSMCs. Therefore, we suggest that the anti-inflammatory properties of CWE, such as the inhibition of the expression of VCAM-1 and ICAM-1, and the reduction in monocyte adhesion to VSMCs, were mainly exerted by 2 types of acetophenones, *p*-hydroxyacetophenone and Cyn A. To clarify our hypothesis, we examined the amounts of the 2 acetophenones in the CWE we used. We found that CWE contained approximately 3.8 mg/g of *p*-hydroxyacetophenone and 21.0 mg/g of Cyn A, respectively.

We investigated the mechanisms through which CWE exerts beneficial effects on the prevention of vascular inflammation. We identified the 2 bioactive components of CWE, *p*-hydroxyacetophenone and Cyn A. These results suggest that the root of *C. wilfordii* and/or its active components may have potential application in the prevention of atherosclerosis and vascular inflammatory diseases.

## Abbreviations

CWE: C*ynanchum wilfordii* ethanol extract
TNF-α: tumor necrosis factor-α
HASMCs: human aortic smooth muscle cells
ICAM-1: intercellular adhesion molecule-1
VCAM-1: vascular cell adhesion molecule-1
NF-κB: nuclear factor-κB
2,4-DHA: 2,4-dihydroxyacetophenone
2,5-DHA: 2,5-dihydroxyacetophenone
*p*-HA: *p*-hydroxyacetophenone
Cyn A: cynandione A
